# Comparing Springtime Ice-Algal Chlorophyll *a* and Physical Properties of Multi-Year and First-Year Sea Ice from the Lincoln Sea

**DOI:** 10.1371/journal.pone.0122418

**Published:** 2015-04-22

**Authors:** Benjamin A. Lange, Christine Michel, Justin F. Beckers, J. Alec Casey, Hauke Flores, Ido Hatam, Guillaume Meisterhans, Andrea Niemi, Christian Haas

**Affiliations:** 1 Polar Biological Oceanography, Alfred-Wegener-Institut Helmholtz-Zentrum für Polar- und Meeresforschung, Bremerhaven, Germany; 2 University of Hamburg, Centre for Natural History (CeNak), Zoological Museum, Biocenter Grindel, Hamburg, Germany; 3 Department of Earth and Atmospheric Sciences, University of Alberta, Edmonton, Alberta, Canada; 4 Freshwater Institute, Fisheries and Oceans Canada, Winnipeg, Manitoba, Canada; 5 Department of Biological Sciences, University of Alberta, Edmonton, Alberta, Canada; 6 Department of Earth and Space Sciences and Engineering, York University, Toronto, Ontario, Canada; Laval University, CANADA

## Abstract

With near-complete replacement of Arctic multi-year ice (MYI) by first-year ice (FYI) predicted to occur within this century, it remains uncertain how the loss of MYI will impact the abundance and distribution of sea ice associated algae. In this study we compare the chlorophyll *a* (chl *a*) concentrations and physical properties of MYI and FYI from the Lincoln Sea during 3 spring seasons (2010-2012). Cores were analysed for texture, salinity, and chl *a*. We identified annual growth layers for 7 of 11 MYI cores and found no significant differences in chl *a* concentration between the bottom first-year-ice portions of MYI, upper old-ice portions of MYI, and FYI cores. Overall, the maximum chl *a* concentrations were observed at the bottom of young FYI. However, there were no significant differences in chl *a* concentrations between MYI and FYI. This suggests little or no change in algal biomass with a shift from MYI to FYI and that the spatial extent and regional variability of refrozen leads and younger FYI will likely be key factors governing future changes in Arctic sea ice algal biomass. Bottom-integrated chl *a* concentrations showed negative logistic relationships with snow depth and bulk (snow plus ice) integrated extinction coefficients; indicating a strong influence of snow cover in controlling bottom ice algal biomass. The maximum bottom MYI chl *a* concentration was observed in a hummock, representing the thickest ice with lowest snow depth of this study. Hence, in this and other studies MYI chl *a* biomass may be under-estimated due to an under-representation of thick MYI (e.g., hummocks), which typically have a relatively thin snowpack allowing for increased light transmission. Therefore, we suggest the on-going loss of MYI in the Arctic Ocean may have a larger impact on ice–associated production than generally assumed.

## Introduction

Arctic first-year sea ice (FYI), from lower latitude and shelf regions, is generally more productive than multi-year ice (MYI), which leads to the assumption that a replacement of MYI by FYI will result in an overall increase of sea ice algal biomass. Arctic sea ice has already undergone a dramatic reduction of MYI with pronounced losses of the oldest and thickest MYI [1–3]. In September 2012, a new record Arctic sea ice extent minimum was set, far exceeding the previous record minimum of 2007, which was itself a remarkable decline from previous years [4,5]. The decline of summer sea ice has occurred concurrently with an increase in duration of the melt season and changes in the timing of melt onset and freeze-up [5–8]. These findings in conjunction with climate-model simulations [9–12] demonstrate that continued Arctic warming and declining Arctic sea ice, with the replacement of MYI by FYI, is likely to continue unabated into the future, having profound consequences for climate feed-backs, physical ocean processes, ecosystem linkages, and Arctic biodiversity [5,13].

The rapid loss of sea ice represents an equally rapid change in habitat for sea ice algae, protists, and ice-associated fauna. Sea ice algae represent an important and high quality food source, directly or indirectly, for many key organisms found in polar regions (e.g. copepods, amphipods, sea birds, polar cod, seals, polar bears; [14–17]). In the Arctic, the timing of ice algal growth is important for the reproduction and growth of key grazing zooplankton species, such as copepods [18,19]. Ice algae provide food for pelagic grazers or may sink at the time of ice melt to the benthos where they are consumed by benthic communities or sequestered into the sediments (e.g., [20]). Therefore, changes in ice algal biomass and distribution are expected to strongly impact Arctic food webs and the Arctic carbon cycle, which can have cascading impacts on global-scale ecological interactions and the global carbon budget.

Sea ice decline, thinning of Arctic sea ice, and the loss of MYI have resulted in reduced Arctic-wide sea ice albedo [21] and more light reaching the under-ice environment in summer [22]. Such conditions have been suggested to be conducive to the development of under ice phytoplankton blooms, which may become more prominent in the future [23]. Reductions in sea ice thickness and extent have also been linked to increases in primary production in coastal shelf regions [24,25]. However, current and future estimates for primary production, including ice algal and phytoplankton growth, in the central Arctic Ocean remain uncertain. Even with increased light availability, primary production may be limited by nutrient supply, resulting in part from increased surface water stratification [26].

The development of sea ice algal communities is influenced by sea ice microstructure (e.g., salinity and temperature which influence permeability), nutrient supply, and transmitted irradiance (see recent review in [27]). During spring, the main influences on under-ice irradiance are the snow depth distribution, with snow extinction coefficients between 4 to 80 m^-1^[28–31], and ice thickness, to a lesser extent, with extinction coefficients between 0.8 to 1.55 m^-1^[28,32–34]. Initial growth of sea ice algae, during early spring, is primarily controlled by the snow distribution, which is typically evident by a negative relationship between chlorophyll *a* (chl *a*) and snow depth (e.g., [35,36]). During the progression of melt, light transmission increases due to changes in the optical properties of snow and ice [34,37]. Consequently, ice algal growth increases and shifts to a more nutrient-limited system, which can be accompanied by a combination of other limiting factors such as: self-shading, diurnal light patterns, or ice ablation [38–40]. In some instances when light transmission increases faster than algal communities can adapt, the increased light field can reduce activity and biomass of algal communities due to photo-inhibition [41,42]. Ice algal growth and the bloom period are terminated during advanced and rapid melt [40].

Many studies have characterized the relationship between snow depth, transmitted irradiance, and chl *a* for FYI (e.g., [35,36]), however, little is known about these relationships for MYI. In general, the large majority of studies dealing with ice algae or chl *a* biomass focus on landfast FYI (e.g., [43–47], see also summary in [48]). These limitations stem from the logistical constraints of sampling within the Arctic Ocean, particularly within regions dominated by MYI.

A summary of studies concerning Arctic MYI chl *a* biomass (Table 1) reveals the need for more recent observations within MYI-dominated regions during the onset of algal growth (e.g., April to May). The available MYI studies are all currently over nine years old with the majority covering the summer season (Table 1). The four studies conducted during the winter-spring transition were conducted within the Bering Sea, Greenland Sea, Fram Strait and Beaufort Sea (Table 1) leaving a large portion of the MYI covered Arctic with no observations during this transitional period. Of all these MYI studies (Table 1), none characterize the chl *a*-snow depth relationship or provide a detailed comparison between FYI and MYI chl *a* biomass for the same region, which could provide insight into a future Arctic Ocean with little or no MYI. Most of the MYI studies listed in Table 1, except for the three most recent studies (e.g., [49–51]), were conducted in a different Arctic system when the melt season was shorter [7], temperatures were colder [52], sea ice was thicker [3], and MYI dominated [1]. Thus, it may be stated that our current understanding of Arctic sea ice algae and chl *a* biomass is based on observations with limited spatial and temporal coverage from regions that have experienced pronounced changes. As a result, there is a need for additional MYI chlorophyll observations to fill important spatial, temporal and seasonal (i.e., spring period) gaps.

**Table 1:** Summary of relevant studies on Arctic MYI chlorophyll *a* biomass.

Region	Season	Year(s)	Study
Beaufort-Chukchi Seas	Year-round	1997–1998	Melnikov et al. (2002) [77]*
Fram Strait	Winter	1993	Thomas et al. (1995) [76]
Fram Strait	Winter-Spring & Summer	2002 & 2003	Schünemann and Werner(2005) [49]*
Greenland Sea & FramStrait	Spring-Summer	1997	Werner and Gradinger(2002) [79]*
Bering Sea	Spring	<1974	McRoy and Goering (1974)[78]
Central Arctic Ocean	Summer	1991 & 1994	Gradinger (1999) [81];Gosselin et al. (1997) [92]
Beaufort-Chukchi Seas	Summer	2002 & 20032005	Gradinger et al. (2005) [51]Gradinger et al. (2010) [50]
Greenland Sea	Summer	1994 & 1995	Gradinger et al. (1999) [95];Werner and Zhang (2002)[79]*
Barents Sea	Summer	1993	Gradinger and Zhang (1997) [96]

*studies conducted during multiple seasons.

The north-eastern coast of Canada, including the Lincoln Sea, represents an important region as it is home to some of the oldest and thickest ice in the Arctic and will likely be one of the last remaining refuges for MYI in the future [1,5,53,54]. Despite the importance of this region, we are aware of only two sea ice biogeochemical studies in the Lincoln Sea, characterizing microbial communities [55] and denitrification [56].The Lincoln Sea is one of the last remaining places where baseline observations of older (>3 years) MYI biogeochemical properties are possible and a comparison between MYI and FYI would provide much needed insight into the future of Arctic marine ecosystems. Based on the limited studies of MYI and the spatial bias of FYI studies, it is difficult to estimate how Arctic sea ice algal biomass will change with a shift to a FYI dominated system.

The main goal of our study was to determine if FYI has, or has the potential for, higher chl *a* biomass than MYI in the Lincoln Sea and discuss the implications of our results in the context of a future Arctic with little or no MYI. We address our scientific question first by providing detailed analyses of the physical properties and chl *a* concentrations of sea ice (both MYI and FYI) in three consecutive spring seasons, from a region where no similar studies have been reported. Then we evaluate potential differences of ice-algal chl *a* concentrations and biophysical sea ice properties between ice types, ice ages, and texture classes. Lastly, we investigate the relationship between sea ice chl *a* concentrations and environmental properties, such as snow depth, sea ice structure, and light availability.

## Materials and Methods

### Study region

In order to conduct this research project in accordance with regulations set forth by the governing agencies responsible for the study region, all relevant research licenses and permissions were acquired from the Nunavut Research Institute (License numbers: 02–075 10R-M; 02–108 11R-M; 02 012 12R-M) and Nunavut Impact Review Board (Screening Decision Report 08YN057).

**Fig 1:**
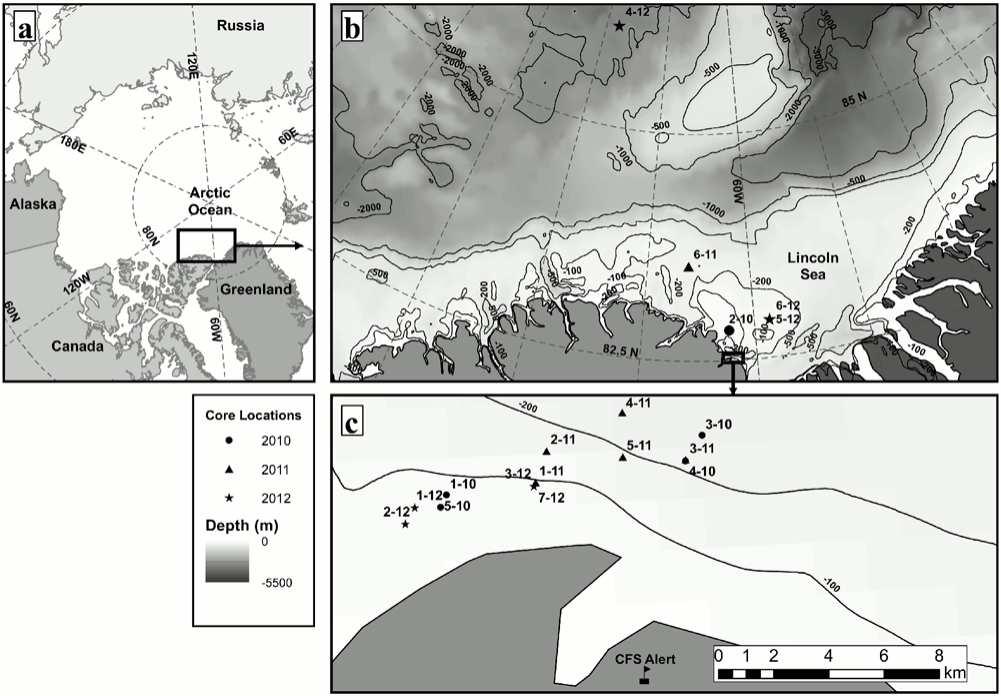
Overview Maps of the study region and ice coring sites. a) Map of the Arctic Ocean with an outline of the study region. b) Map of the Lincoln Sea and neighboring regions. Drifting ice sites (pack ice), ocean bathymetry, and an outline of landfast ice sites are indicated. c) Map of landfast ice coring sites, immediately offshore from CFS Alert.

The Lincoln Sea (Fig 1) has been called the “Last Ice Area” by the World Wildlife Fund based on recommendations from Arctic Council Assessments, indicating that the Lincoln Sea requires specific attention and research [57,58]. Most studies in this region focus on physical properties (of the MYI) and have documented a slight decline in modal ice thickness since 2004 from between 4.0 and 4.5 m (pre-2008 observations) to 3.5 m (post-2008 observations), which is likely the result of less old ice along the northern coast of Canada [5,54].

The Lincoln Sea is a dynamic area due to interaction with, and exchange of, sea ice with the Arctic Ocean. The Lincoln Sea ice cover is comprised of immobile landfast coastal sea ice at the southern edges and mobile pack ice at its northern extent. The landfast ice consists primarily of consolidated pack ice with smaller amounts of FYI forming in the interstitial space during freeze-up. The division between landfast ice and pack ice is not a distinct line but rather a transitional region that can be characterized by ice with limited mobility due to geographic barriers and the intermittent nature of ice export to the south through Nares Strait. Sea ice in the Lincoln Sea typically comes from the Central Arctic Ocean transported by the Beaufort Gyre and Transpolar Drift circulation patterns [59,60]. However, the origin of sea ice in the Lincoln Sea is uncertain because ice ages are typically between 2 to 5 years, with a decreasing proportion of >5 year old ice [1]. This means that ice in this region could have originated from anywhere in the Arctic Ocean.

### Sampling

Sampling was conducted during spring in the first two weeks of May 2010, 2011 and 2012 in the Lincoln Sea. One site was located north of the Lincoln Sea (Fig 1). Sea ice cores were taken at a total of 18 sites: 11 MYI sites (4 in 2010, 4 in 2011 and 3 in 2012), and 7 FYI sites (1 in 2010, 2 in 2011 and 4 in 2012), including landfast ice and mobile pack ice (Fig 1). Landfast ice sites were visited by snowmobile, and pack ice sites were visited by helicopter or Twin Otter aircrafts.

During this time of year, when temperatures are typically below -10°C and the low salinity MYI is very hard, coring thicker than ~3.5 m becomes exponentially more difficult. We therefore chose relatively level sites that we knew were below ~3.5 m based on pre-drilled 2 inch auger thickness holes.

At each site three ice cores were extracted within 1 m of each other using a 9 cm inner diameter ice corer (Kovacs Enterprise Mark II) and stored in sterile U-Line bags. One core was sampled for texture and bulk salinity (“Texture core”), one core for chlorophyll *a* (“chl *a* core”) and one to two cores for microbial genetics (genetic methodology/protocol and results from one MYI site “1–11” presented elsewhere, see [55]). Due to small discrepancies between core lengths at the same site, texture core lengths were adjusted to correspond to the chl *a* core length by linearly interpolating each depth value (e.g. texture class, bulk salinity, temperature and brine volume) of the texture cores proportionally. All cores were transported from the field back to the Canadian Forces Station (CFS) Alert, Nunavut, Canada (82.5°N, 62.5°W) and stored at -15 to -20°C in the dark.

### On-site measurements

At each core location, snow depth (here after referred to as core-location-snow-depth), freeboard and core length were measured. These measurements represent the local conditions (i.e., one single point) at sampling locations, which should not be confused with the larger-scale snow depth and ice thickness survey measurements. Internal ice temperatures were measured on texture cores by drilling holes and inserting a thermometer (Testo 720) immediately after core extraction. Temperatures were measured from surface to bottom at intervals of 0.1 m (cores: 1–10, 3–10 and 4–10) and 0.5 m (core 5–10). In 2012, only ice surface temperature (depth 0.1 m) was measured. For these cores the internal ice temperatures were linearly interpolated between the surface and assumed (theoretical) bottom temperature of -1.78°C with typical surface water salinities in the Lincoln Sea of ~32 [61]. During the study period daily temperature variation within the ice was minimal and based on the measured temperature profiles a linear relationship with ice depth demonstrated a good fit (R^2^ = 0.94). Brine volume estimates were calculated for cores with temperature measurements using equations in [62]. Brine volume values are reported in parts per thousand (ppt).

Snow depth and ice thickness surveys were conducted along transects adjacent to each coring site. These measurements represent the larger-scale characteristics of the sampled ice floes, which should not be confused with the point measurements: core-location-snow-depth and core length. Snow depth was measured using a metal probe at 1 or 10 m intervals and ice thickness was measured in 2 inch augers holes drilled in the ice at 10 m intervals. The length of each transect and number of measurements were dependent on ice type and time constraints (range = 0 to 400 m, mean = 100 m). Snow density measurements were calculated for 5 snow samples collected, at site 2–10, using an Adirondack snow sampler. Additional density values from the same study region were acquired during the CryoSat Validation Experiment (CryoVEx: 11 to 18 April, 2011[63]).

Air temperature data were provided by the Environment Canada weather station located on shore at CFS Alert, Nunavut. Mean daily air temperatures during the study were on average -12.5°C (2010 to 2012 combined), with a range between -20.3 to -7.4°C, and a maximum temperature of -3.1°C. Downwelling total solar irradiance measurements representative of the sampling area were also measured at the nearby CFS Alert weather station. Mean and range of values were calculated for the period May 1–11, 2010 to 2012 (mean = 984, range = 213 to 2313 μmol photons m^-2^ s^-1^; data acquired from NOAA / ESRL / GMD / GRAD, the GMD-Radiation Group, ftp://aftp.cmdl.noaa.gov/data/radiation/baseline/alt/).

### Texture cores

**Fig 2:**
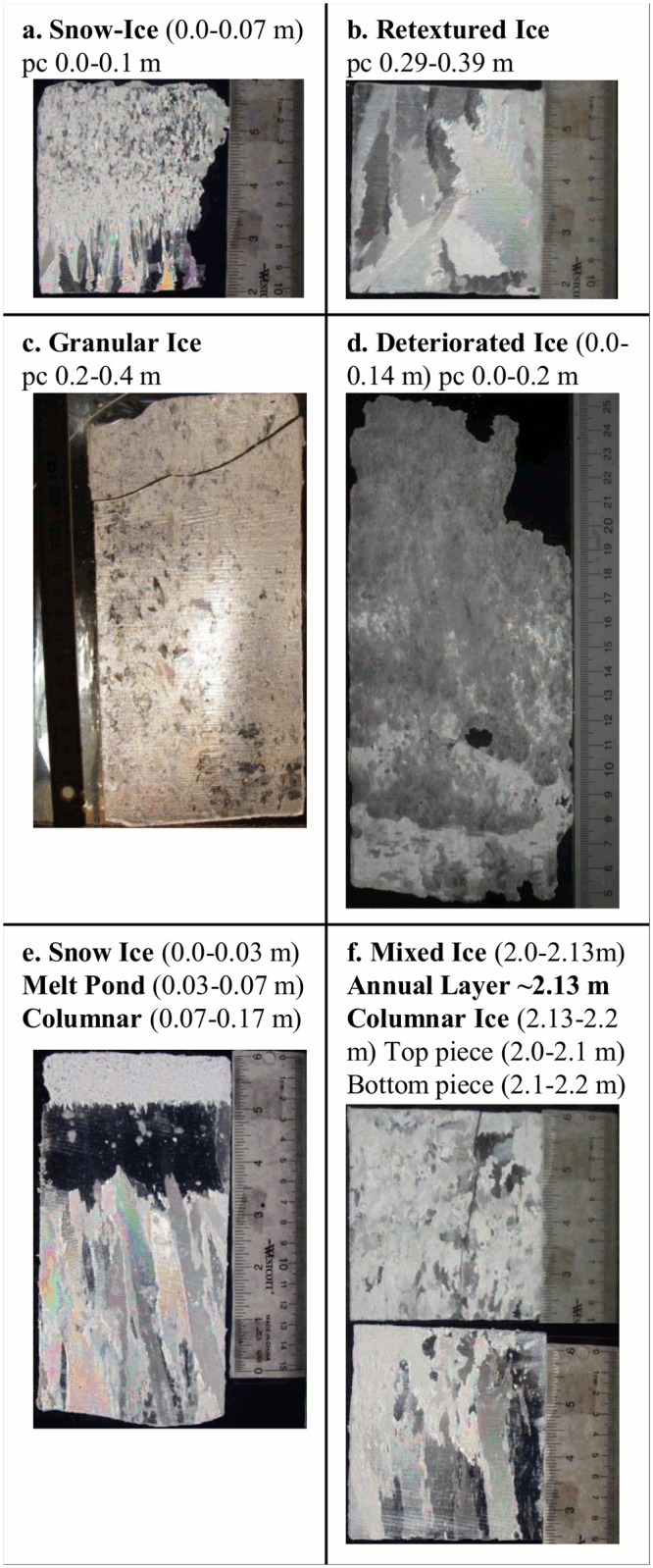
Cross polarized imagery of ice core thin sections showing different ice types. a) core 7–12; b) core 7–12; c) core 2–10; d) core 3–11; e) core 1–12; and f) core 7–12.

Texture analysis was conducted at ~ -15°C. Texture cores were cut into 0.10 to 0.15 m vertical sections that were further cut into vertical thick sections ~ <5 mm thin, using an electric band saw. For each section, the ice remaining after cutting was put into plastic containers, melted and analyzed for bulk salinity using a salinometer (WTW 3300i). Bulk salinities are reported in parts per thousand (ppt). Thick sections were imaged under crossed polarizers. Analysis of the images provided a stratigraphic description of each ice core by identifying different ice texture classes and the boundaries between classes. Here we divided ice types into 7 texture classes based on grain structure and appearance, following classification systems outlined in [64–67](Table 2 and Fig 2). For each section of the chl *a* cores, the dominant texture class (i.e. the texture class with the highest areal coverage) from the corresponding texture core was assigned. For surface pieces the uppermost texture class was always assigned to the section because deteriorated ice and snow-ice are distinct layers and only located at the surface.

**Table 2:** Description of each sea ice texture class.

Ice Class	Description
Snow-Ice	Looks like granular but is clear in un-polarized images. Forms during flooding or with presence liquid water and snow near freezing and forms small granular crystals during rapid freezing.
Melt Pond	Fresh water, clear in appearance, at or very near the surface of the ice. Sometimes overlaid by snow-ice.
Retextured	Clear ice with unusual crystals or very large crystals, forms near surface below water level
Deteriorated	Transformed columnar or mixed ice with large brine or air pockets, near the surface usually above water level.
Granular	Consolidation of frazil ice usually near the surface (typically with mixed layer underneath). This can occur within MYI and is evidence of super-cooling, turbulent water and/or presence of adjacent re-freezing lead which creates conditions for rapid freezing and formation of frazil ice.
Mixed col./gran.	Mixture of congelation and granular ice. This class also includes intermediate congelation/granular ice because they are difficult to distinguish.
Columnar	Elongated crystals

In MYI cores, we identified the previous year’s annual layer in 7 cores by identifying peaks in bulk salinity profiles that corresponded with changes in crystal structure at the same depth, as described in [68,69]. Accordingly, MYI cores were divided into two groups: the sections above the annual layer (older ice) were classified as multi-year (MY) and sections below the annual layer (new ice) were classified as first-year (FY). Therefore, in later analyses ice type categories comprise MYI cores and FYI cores, whereas ice age categories comprise MY (multi-year sections of MYI cores), FY (first-year ice sections of MYI cores) and FYI (first-year ice cores). MY portions represent ice that has survived at least one summer. The FY portions represent ice that has grown under the MY portions and typically begins to form during freeze-up in September-October [7]. In general FYI and FY represent ice of similar ages (e.g. <1 year), however, FYI can represent ice that started to form either during freeze-up or at a later stage as open water leads form and refreeze. FY also grows slower than FYI due to increased thermal insulation of the thicker ice (MY) and snow layer above it.

### Chlorophyll *a* cores

In order to minimize any potential influences on the chl *a* measurements, these cores were always stored below -15°C in the dark, for a maximum of nine days. The cores were then shipped, via air at a maximum temperature of -10°C in the dark, to Resolute Bay, Nunavut, where they were stored below -20°C in the dark for 1 to 2 days. Cores were cut using an electric band saw (sterilized with 95% ethanol) in a -20°C walk-in freezer. Cores were cut into 10 cm sections except for end pieces (range: 0.09 to 0.17 m), placed in sterile Whirl-Pack (NASCO) bags and melted in the dark. End pieces for 2010 and 2012 samples refer to the ice-air interface. The 2011 cores were cut in the opposite direction, therefore these end pieces refer to the ice-water interface. Core sections were melted without the addition of filtered sea water (FSW) because we were also measuring dissolved constituents (i.e. DOC, nutrients; data not presented here) in the core sections. It has been shown that for common biological analyses (e.g., chlorophyll *a* concentrations) melting without the addition of FSW is an acceptable procedure [70].

Chl *a* concentrations were determined on sub-samples filtered onto Whatman GF/F filters, after 24 h extraction in 90% acetone at 4°C in the dark, using a 10AU Tuner Design fluorometer calibrated with pure chlorophyll extract from *Anacystis nidulans* (Sigma; [71]). Chl *a* concentrations were determined using equations from [71] and corresponding instrument calibration coefficients. Chl *a* concentrations are expressed volumetrically (mg m^-3^) or are vertically integrated (mg m^-2^) for the bottom 0.2 m core sections (hereafter referred to as bottom-integrated), age class core sections, or total core length.

### Statistical analyses

Initial data exploration demonstrated that the distributions of chl *a* data were highly skewed. To achieve the normal distribution patterns required for parametric statistical analyses, log-transformations were applied to the chl *a* data. Two-sample t-tests were conducted to determine the effect of ice type (MYI & FYI) on the chl *a* concentrations of the sea ice. Variance analyses (ANOVAs) were conducted in order to determine the effect of ice age class (MY, FY & FYI); year and texture class on the chl *a* concentrations of the ice. Post-hoc tests (Tukey’s HSD) were conducted when both parametric ANOVA (transformed data) and non-parametric Kruskal-Wallis (non-transformed data) analyses showed significant differences.

To investigate the potential influence of snow depth and sea ice optical properties on bottom-integrated chl *a* concentrations a logistic regression model was applied. A logistic regression was used in order to identify potential critical values (inflection point) of the independent variables (e.g., snow depth and bulk integrated extinction coefficients) that could indicate a threshold value for optimal algal growth. Logistic regression analysis required values of the dependent variable in the range 0 to 1.Therefore, bottom-integrated chl *a* values were normalized to the range 0 to 1 by dividing each chl *a* value (y_i_) by the maximum value for all cores (y_max_). The relationship between bottom-integrated chl *a* concentrations and bulk integrated extinction coefficients, for visible radiation, was analyzed in a similar manner. For all calculations we used extinction coefficients for snow: k_s_ = 20.0 m^-1^ [28]; MYI: k_m_ = 1.55 m^-1^; and FYI: k_f_ = 1.45 m^-1^ [32]. The value of k_s_, used here, was chosen from a table of k_s_ values [28] based on a corresponding snow density comparable to measured values for our study region between 260 to 281 kg m^-3^ (see section Physical properties). The values of k_s_, k_m_, and k_f_ were integrated over the depth of the corresponding ice and snow layers for each core site resulting in “integrated extinction coefficients” (dimensionless), i.e., a value for each snow and ice layer at each site. The “bulk (snow plus ice) integrated extinction coefficient” is simply the sum of the integrated extinction coefficients for snow and ice (also dimensionless), i.e., one value for each core site. In the resulting bulk integrated extinction coefficients, larger values mean shallower penetration of light.

Results are reported as arithmetic mean ± one standard deviation (μ̂ ± 1σ).

All statistical analyses were conducted with the R software package v-2.15.2 [72].

## Results

### Physical properties

Based on site-averaged drill hole thickness measurements, i.e., characterizing the larger scale ice properties of the sampled floes, MYI sites were more than twice as thick (3.28 ± 0.56 m) as FYI sites (1.42 ± 0.42 m). Mean MYI core length (2.62 ± 0.24 m), i.e., only characterizing local ice properties of core sampling locations, was also nearly twice as thick as FYI (1.39 ± 0.52 m), with higher variability in FYI core lengths. FYI sites represented two different kinds of ice: ice that formed during the fall when the landfast ice consolidated (i.e., older and thicker FYI with more snow); and ice that formed later in mobile ice when open leads formed and then refroze (i.e., younger and thinner ice with typically less snow).

Site-averaged snow depth at MYI sites (0.39 ± 0.10 m), in general, was thicker than at FYI sites (0.26 ± 0.15 m). Although MYI had lower variability between site-averaged snow depth values, FYI had lower variability when considering each site individually. This was illustrated by the mean of site standard deviations for FYI snow depth: 0.08 m, compared to MYI: 0.17 m. Mean snow density at site 2–10 (Fig 1) was 260 ± 0.03 kg m^-3^ (n = 5) and during CryoVex 2011 was 281 ± 0.7 kg m^-3^ (n = 11 [63]).

There was no significant inter-annual difference in the mean physical properties (e.g. snow depth and ice thickness) of FYI or MYI (ANOVA, p>0.05). For FYI, there were significant positive relationships between core-location-snow-depth and ice core length (R^2^ = 0.56, p = 0.05, n = 7), and between snow depth and mean ice thickness survey measurements (R^2^ = 0.66, p<0.05, n = 7). For MYI, there was an inverse relationship between snow depth and ice core length (R^2^ = 0.51, p = 0.01, n = 11), and no significant relationship between snow depth and mean ice thickness survey measurements (R^2^ = 0.12, p = 0.3, n = 11).

Two FYI cores were exceptional (3–10 and 6–12; Table 3), exhibiting considerably lower ice thickness, snow depth and core length than the other FYI cores analyzed in this study. These 2 sites were determined to be refrozen leads that correspond to younger FYI that formed later in the season. Although core 4–11 core length was not exceptionally low, survey measurements indicated site 4–11 had the thinnest ice and snow pack (excluding sites 3–10 and 6–12; Table 3). The location was also at the edge of the landfast ice where unstable ice is likely even after freeze-up, therefore was also considered to be a younger FYI site, although older than sites 3–10 and 6–12.

**Table 3:** Summary of physical data for each core and core site.

Site Overview						Core Location				Ice Thickness Surveys					Snow Depth Surveys				Texture Class Lengths						
Year	Type	id	lat	lon	bath.	Core	Snow	Sal	BV	*μ̂*	σ	d	N	fb	*μ̂*	σ	d	N	col	mx	gran	retex	mp	s-i	det
2010	FYI	3–10	82.6	-62.0	257	0.93	0.09	6.1	189.4	0.93	-	25	4	0.07	0.08	0.0	45	10	0.88	0.03	0.02	-	-	-	-
2010	MYI	1–10	82.5	-62.6	91	2.23	0.42	3.1	88.1	3.07	0.9	100	6	0.13	0.37	0.1	100	6	0.67	1.40	-	-	0.16	-	-
	MYI	2–10	82.8	-62.3	95	2.80	0.32	3.4	-	3.49	0.4	200	9	0.23	0.33	0.2	200	42	1.94	0.19	0.27	-	0.22	0.16	-
	MYI	4–10	82.6	-62.0	199	2.54	0.50	2.0	133.0	2.45	-	0	1	0.09	0.50	-	0	1	0.73	1.00	0.72	-	-	-	0.10
	MYI	5–10	82.5	-62.6	90	3.11	0.05	2.1	73.3	3.10	0.7	200	9	0.32	0.22	0.2	200	41	0.71	0.07	2.27	-	-	-	0.05
	*μ̂*	*-*	*-*	*-*	*119*	*2*.*67*	*0*.*32*	*2*.*6*	*98*.*2*	*3*.*03*	*0*.*7*	*125*	*6*	*0*.*19*	*0*.*36*	*0*.*2*	*125*	*23*	*1*.*01*	*0*.*66*	*1*.*09*	*-*	*0*.*19*	*0*.*16*	*0*.*08*
2011	FYI	2–11	82.6	-62.3	189	1.60	0.38	4.5	-	1.67	-	0	3	0.04	0.38	-	0	1	0.23	0.06	1.32	-	-	-	-
	FYI	4–11	82.6	-62.2	242	1.52	0.17	5.5	-	1.31	0.2	40	5	0.07	0.21	-	40	5	1.40	0.11	0.01	-	-	-	-
	*μ̂*	*-*	*-*	*-*	*215*	*1*.*56*	*0*.*28*	*5*.*0*	*-*	*1*.*49*	*0*.*2*	*20*	*4*	*0*.*06*	*0*.*30*	*-*	*20*	*3*	*0*.*82*	*0*.*08*	*0*.*66*	*-*	*-*	*-*	*-*
2011	MYI	1–11	82.5	-62.4	100	2.41	0.55	3.5	-	3.85	1.1	100	10	0.29	0.36	0.2	100	10	1.51	0.24	-	0.18	0.39	0.09	-
	MYI	3–11	82.6	-62.0	199	2.96	0.32	2.1	-	2.80	0.6	100	12	0.17	0.52	0.2	100	12	1.45	0.52	0.81	-	-	-	0.17
	MYI	5–11	82.6	-62.2	159	2.53	0.32	3.9	-	4.48	1.4	100	9	0.33	0.39	0.2	100	9	1.20	-	-	1.01	0.26	0.06	-
	MYI	6–11	83.5	-66.0	260	2.59	0.30	2.4	-	3.02	0.8	100	10	0.17	0.55	0.2	100	10	2.35	0.13	-	-	-	-	0.11
	*μ̂*	*-*	*-*	*-*	*180*	*2*.*62*	*0*.*37*	*3*.*0*	*-*	*3*.*54*	*1*.*0*	*100*	*10*	*0*.*24*	*0*.*46*	*0*.*2*	*100*	*10*	*1*.*63*	*0*.*30*	*0*.*81*	*0*.*59*	*0*.*33*	*0*.*08*	*0*.*14*
2012	FYI	2–12	82.5	-62.7	57	1.77	0.20	4.4	90.4	2.00	-	100	4	0.11	0.28	0.1	100	101	1.42	0.07	0.28	-	-	-	-
	FYI	3–12	82.5	-62.4	99	1.34	0.33	3.7	-	1.51	0.1	20	2	0.05	0.33	0.1	20	21	1.25	0.04	0.05	-	-	-	-
	FYI	4–12	86.1	-78.1	2156	1.77	0.47	3.1	87.7	1.68	-	0	1	0.06	0.47	-	0	1	1.42	0.34		-	-	-	-
	FYI	6–12	82.9	-58.6	125	0.83	0.04	5.9	141.4	0.83	0.1	40	3	0.00	0.07	0.0	40	41	0.77	0.05	0.01	-	-	-	-
	*μ̂*	*-*	*-*	*-*	*609*	*1*.*43*	*0*.*26*	*4*.*3*	*106*.*5*	*1*.*51*	*0*.*1*	*40*	*3*	*0*.*05*	*0*.*29*	*0*.*1*	*40*	*41*	*1*.*21*	*0*.*13*	*0*.*11*	*-*	*-*	*-*	*-*
2012	MYI	1–12	82.5	-62.7	86	2.67	0.47	3.3	116.5	3.69	0.9	400	11	0.24	0.39	0.2	400	401	1.79	0.81	-	-	0.04	0.02	-
	MYI	5–12	82.9	-58.6	125	2.57	0.31	2.0	115.9	3.23	1.3	120	7	0.29	0.30	0.1	140	140	2.31	0.19	-	-	-	-	0.06
	MYI	7–12	82.5	-62.4	99	2.46	0.60	3.6	184.0	2.90	0.5	200	10	0.20	0.40	0.2	200	221	1.69	0.29	-	0.30	0.11	0.07	-
	*μ̂*	*-*	*-*	*-*	*103*	*2*.*56*	*0*.*46*	*3*.*0*	*138*.*8*	*3*.*27*	*0*.*9*	*240*	*9*	*0*.*24*	*0*.*36*	*0*.*2*	*247*	*254*	*1*.*93*	*0*.*43*	*-*	*0*.*30*	*0*.*07*	*0*.*05*	*0*.*06*

All measurements are in meters (m). Abbreviations and symbols: lat = latitude, lon = longitude, bath. = bathymetry, core = core length, snow = core-location-snow-depth, sal = bulk salinity (ppt), BV = core averaged brine volume (ppt), μ̂ = arithmetic mean, σ = one standard deviation, d = distance, N = sample number, fb = mean freeboard, col = columnar,mx = mixed, gran = granular, retex = retextured, mp = melt-pond, s-i = snow-ice, det = deteriorated.

“-”= Not available.

**Fig 3:**
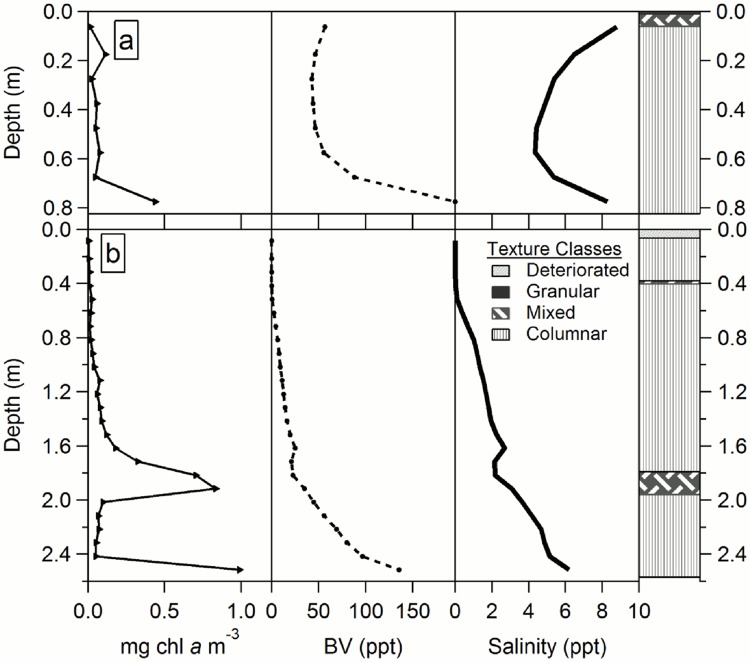
Example vertical profiles for MYI and FYI. Chl *a*, brine volume (BV), bulk salinity, and texture classes for: a) FYI core 6–12 and b) MYI core 5–12.

In all cores, observed internal ice temperatures increased towards the ice-water interface. Surface ice temperatures ranged between -11.5 and -5°C. Bottom ice temperatures were consistently close to the freezing point of sea water (~ -1.78°C; see section On-site measurements). All FYI cores followed typical C-shaped bulk salinity curves (Fig 3a), except for core 4–12 which showed a bulk salinity profile similar to MYI. MYI cores followed typical vertical bulk salinity profiles for MYI with low salinities (0 to 2) near the surface and a general increasing trend towards the bottom ice (Fig 3b). Brine volume also increased with depth in most cores. Only core 1–12 had a brine volume peak at 0.57 m.

**Fig 4:**
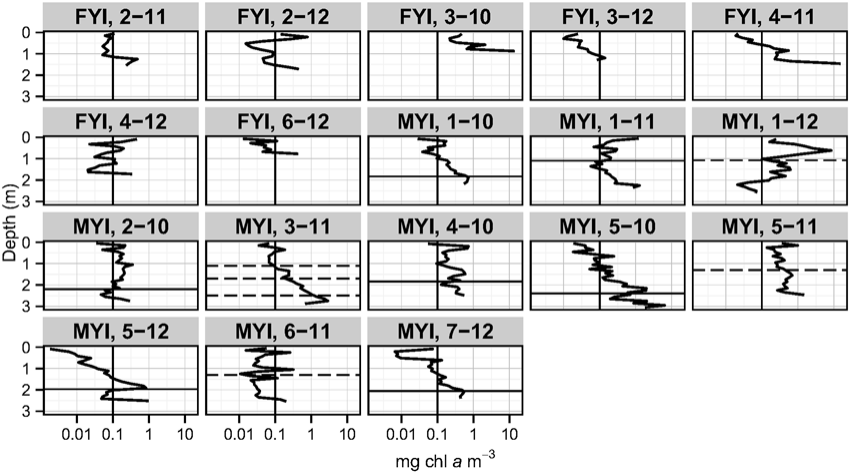
Vertical profiles of chl *a* concentrations for all cores. Solid horizontal lines represent identified annual layers used in the analyses and dashed horizontal lines represent potential annual layers not used in the analyses (x-axis is log scale).

Based on the measured length of each texture class, FYI cores consisted predominantly of columnar ice (76%), with only minor proportions of granular (17%) and mixed (7%) ice. Retextured, melt pond, snow-ice and deteriorated texture classes were not identified in FYI and represented ≤5% of MYI but in some instances represented up to 50% of MYI (e.g., core 5–11; Table 3). MYI cores had a lower proportion of columnar ice (57%), over twice the amount of mixed ice (17%), and approximately equal proportions of granular (14%) ice compared to FYI. Annual growth layers were identified in 7 out of 11 MYI cores (1–10, 2–10, 4–10, 5–10, 1–11, 5–12 and 7–12: Fig 4). The FY portions (i.e., ice below the annual layer) had a mean length of 0.67 ± 0.31 m and the MY portions (i.e., ice above the annual layer) had a mean length of 1.90 ± 0.40 m. Potential annual growth layers were also identified for the remaining 4 MYI cores (1–12, 3–11, 5–11 and 6–11: Fig 4); however, these were not assigned as annual layers in the analysis due to lack of confidence (e.g. presence of multiple layers or lack of correspondence between texture change and bulk salinity peak/change).

A summary of all sea ice physical properties is provided in Table 3.

### Chlorophyll *a*

**Fig 5:**
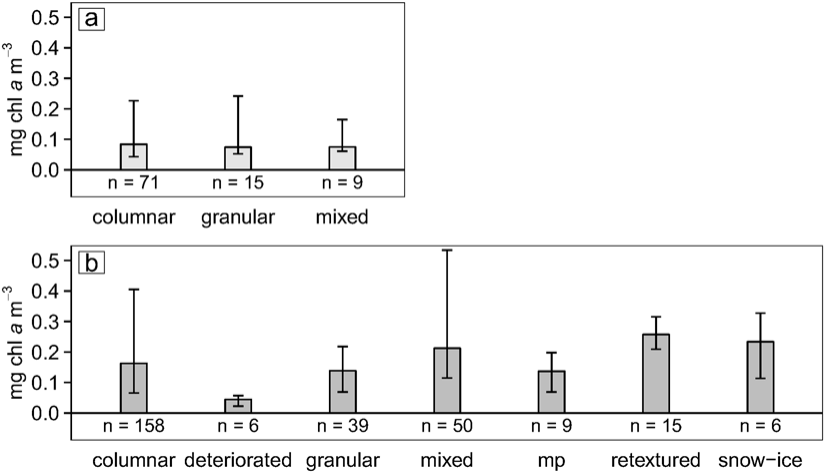
Chl *a* concentrations in each texture class. a) FYI; and b) MYI. Bars show median values with error bars delineating the 25 and 75 percentiles.

All ice cores, with the exception of core 1–12, had chl *a* concentration peaks or maximum values in the bottom sections (Fig 4). Most core sections had chl *a* concentrations <3 mg m^-3^ (Figs 4 and 5). Two FYI cores had chl *a* concentrations >10 mg m^-3^ in the bottom sections (~0.1 m): core 3–10 (14.1 mg m^-3^) and 4–11 (15.4 mg m^-3^; Fig 4). These two FYI cores also corresponded to younger FYI sites (e.g. refrozen lead). Two MYI cores had sections with chl *a* concentrations >5 mg m^-3^: core 5–10 (14.1 mg m^-3^; section midpoint 0.15 m from bottom), which corresponded to a MYI hummock with the lowest core-location-snow-depth, and core 1–12 (8.4 mg m^-3^; section midpoint 0.57 m from surface), which had a brine volume peak at the same depth as the chl *a* peak. In all MYI cores with confirmed annual layers, peaks in chl *a* concentrations closely matched the depths of the annual layers (Figs 3 and 4). Ice cores with re-frozen melt ponds (e.g. 1–10, 1–11, 1–12, 2–10, 5–11 and 7–12) also showed local chl *a* peaks near the surface (Figs 4 and 5). Examples of chl *a*, bulk salinity, and brine volume profiles with coincident texture classes are shown in Fig 3.

### Comparison of ice classes

**Fig 6:**
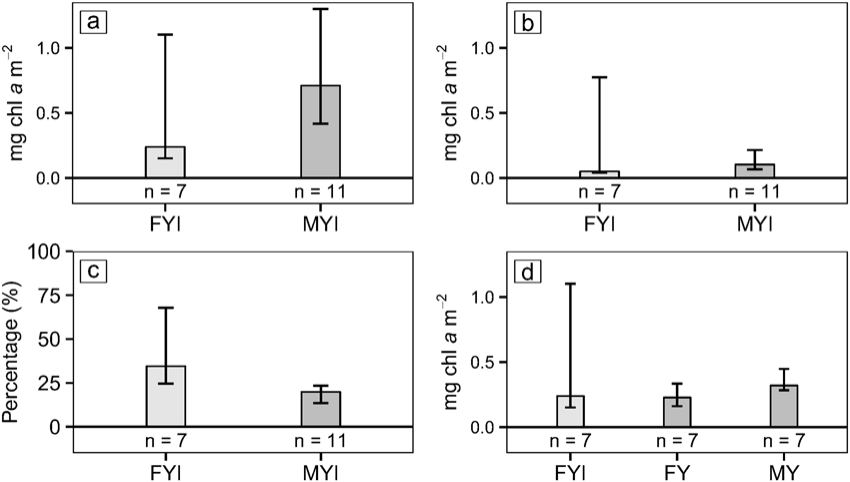
Summary of chl *a* concentrations in different ice types and age portions. Bars show median values with error bars delineating the 25 and 75 percentiles for: a) core-integrated chl *a* in MYI (dark-gray) and FYI (light-gray); b) bottom-integrated chl *a* for MYI and FYI; c) percent of the total chl *a* in the bottom 0.2 m for FYI and MYI; and d) chl *a* integrated over lengths of FYI cores (light-gray) and over age class sections for first-year (FY) and multi-year (MY) portions of MYI (dark-gray).

We found significantly higher bulk salinity values in FYI compared to MYI. MYI cores had higher mean core-integrated chl *a* concentrations (0.93 ± 0.68 mg m^-2^) than FYI (0.71 ± 0.92 mg m^-2^). These differences, however were not statistically significant (t-test, p = 0.1). We also found no significant effect of ice type on volumetric or areal chl *a* concentrations (Fig 6). However, the relative chl *a* concentrations (e.g., fraction of the total core-integrated chl *a*) in the bottom 0.2 m were significantly higher in FYI than in MYI (Fig 6).

One-way ANOVA tests were conducted to compare the effect of ice age (i.e., the upper multi-year portion of multi-year ice [MY]; the bottom first-year portion of multi-year ice [FY] and first-year ice cores [FYI]) on the chl *a* distribution of sea ice. There was no significant effect of the ice age portions on age class-averaged volumetric or areal chl *a* concentrations (Table 4).

**Table 4:** Statistical summary of results comparing chlorophyll *a* between ice age portions (MY, FY and FYI).

Variable	Description	FYI mean ± SD	FY mean ± SD	MY mean ± SD
**chl *a***	mean (mg m^-3^)	0.58 ± 0.83	0.55 ± 0.56	0.21 ± 0.09
	integrated (mg m^-2^)	0.71 ± 0.92	0.36 ± 0.40	0.40 ± 0.20

* Indicates significant test result at p<0.05.

**Fig 7:**
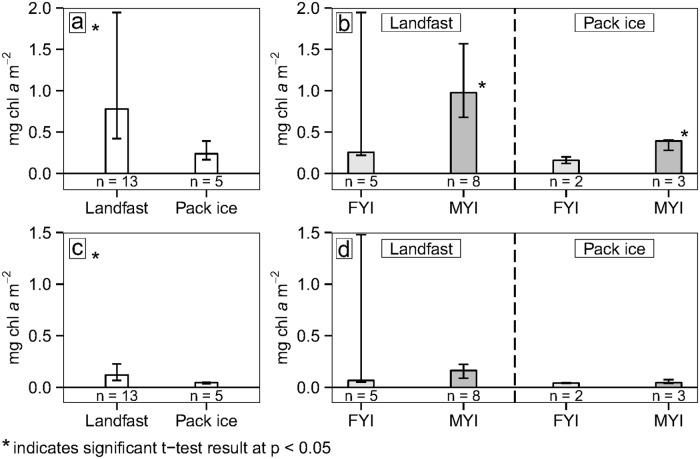
Summary of chl *a* concentrations comparing landfast ice and pack ice. a) Core-integrated chl *a* for all cores (MYI and FYI combined); b) core-integrated chl *a* categorized into FYI (light-gray) and MYI (dark-gray), then by landfast ice (left) and pack ice (right); c) bottom-integrated chl *a* for all cores; and d) bottom-integrated chl *a* categorized into FYI (light-gray) and MYI (dark-gray), then by landfast ice (left) and pack ice (right). Bars show median values with error bars delineating the 25 and 75 percentiles.

When all ice cores were compared, bottom-integrated and core-integrated chl *a* concentrations were significantly higher in landfast ice than in pack ice (Fig 7a and 7c). Multi-year-landfast ice had significantly higher core-integrated chl *a* concentrations than multi-year-pack ice (Fig 7b). No significant differences were observed when we compared first-year-landfast ice to first-year-pack ice, landfast-MYI to landfast-FYI, or pack-MYI to pack-FYI (Table 5; and Fig 7b and 7d).

**Table 5:** Statistical summary of results comparing chlorophyll *a* between ice types (MYI vs. FYI and landfast ice vs. pack ice).

Variable	Description	FYI mean ± SD	MYI mean ± SD
Bulk **salinity (ppt)**	-	*** 4.75 **±** 1.02	***2.85 **±** 0.69
Percent of total **chl *a*** inbottom 0.2 m (%)	-	** 46 **±** 27	** 20 **±** 10
**chl *a*** (bottom 0. 2 m):	mean (mg m^-3^)	2.58 **±** 3.76	0.89 **±** 1.07
	integrated (mg m^-2^)	0.52 **±** 0.75	0.18 **±** 0.21
**chl *a*** (entire core)	mean (mg m^-3^)	0.58 **±** 0.77	0.35 **±** 0.22
	integrated (mg m^-2^)	0.71 **±** 0.85	0.93 **±** 0.64
**chl *a*** (bottom-integrated):	landfast ice (mg m^-2^)	0.70 **±** 0.91	0.22 **±** 0.25
	pack ice (mg m^-2^)	0.04 **±** 0.01	0.06 **±** 0.04
**chl *a*** (core-integrated):	landfast ice (mg m^-2^)	0.92 **±** 1.02	1.16 **±** 0.66
	pack ice (mg m^-2^)	0.16 **±** 0.11	0.32 **±** 0.14
**-**	**-**	**Landfast ice mean ± SD**	**Pack ice mean ± SD**
**chl *a*** (bottom-integrated):	all cores (mg m^-2^)	* 0.41 **±** 0.61	* 0.05 **±** 0.03
	MYI (mg m^-2^)	0.22 **±** 0.25	0.06 **±** 0.04
	FYI (mg m^-2^)	0.70 **±** 0.91	0.04 **±** 0.01
**chl *a*** (core-integrated):	all cores (mg m^-2^)	* 1.07 **±** 0.78	* 0.26 **±** 0.14
	MYI (mg m^-2^)	* 1.16 **±** 0.66	* 0.32 **±** 0.14
	FYI (mg m^-2^)	0.92 **±** 1.02	0.16 **±** 0.11

Note: entire core mean values account for the length of each section in terms of its contribution to the core mean value and therefore can be slightly different from mean values reported in text for core sections. Significant test results indicated by:

* p<0.05;

** p<0.01;

*** p<0.001.

### Relationships between chl *a*, snow depth, and bulk integrated extinction coefficients

**Fig 8:**
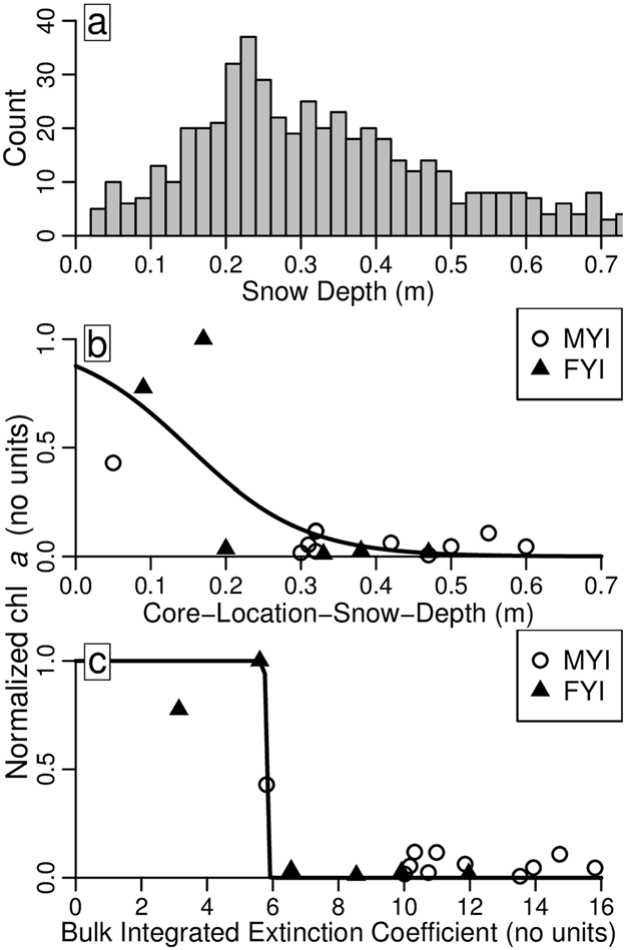
Snow histogram and logistic regressions. a) Histogram of snow depth survey measurements at MYI sites (n = 538). Logistic regressions of normalized bottom-integrated chl *a* concentrations as a function of: b) snow-depth at core locations; and c) bulk (snow plus ice) integrated extinction coefficients.

Logistic regressions were conducted to assess the relationship of normalized bottom-integrated chl *a* concentrations with snow depth and bulk integrated extinction coefficients, respectively (Fig 8). Core 6–12 was excluded from this analysis because it was located on a recently refrozen lead that likely experienced different growth conditions and had different snow properties (e.g., thinnest ice and snowpack compared to the other two young FYI sites 3–10 and 4–11). The logistic regression for snow depth showed a step-wise transition with an inflection point at approximately 0.17 m (Fig 8b). The logistic regression for bulk integrated extinction coefficients shows a more abrupt step-wise transition with an inflection point at a value of 5.8 (Fig 8c).

## Discussion

### Sea ice thickness

MYI in the Lincoln Sea constitutes some of the thickest sea ice remaining in the Arctic Ocean [5,53]. Our total thickness (snow plus ice) survey measurements (MYI: 3.0 to 4.9 m; FYI: 0.9 to 2.3 m) are in agreement with aerial and ground-based total thickness measurements conducted in the same study region, with modal total thickness values for MYI between 3.1 and 5.0 m, and for FYI between 0.9 and 2.0 m [53,54]. With a range of 0.86 to 2.24 m, the total thickness (core length plus core-location-snow-depth) of core sites for FYI was in line with the larger scale survey measurements. MYI core sites, however, demonstrated slightly lower total thickness (core length plus core-location-snow-depth) values (2.65 to 3.28 m) than in the survey measurements. MYI, especially in the Lincoln Sea, has a very broad thickness distribution. Although the total thickness of the MYI cores remained within the broader thickness distribution they do not represent the thicker end of a typical ice distribution in the Lincoln Sea. This sampling bias is due to the increasing difficulty and time required for sampling as ice thickness increases.

### Snow depth

The mean snow depth for MYI of 0.39 m (present study) was representative of the region when compared to the mean snow depth of 0.3 m found by [53]. The controlling factors for snow accumulation and distribution on MYI and FYI, however, were different. When analyzing all FYI sites together, we found a positive relationship between core snow depth and ice core length. This was surprising since on a FYI floe of uniform age a negative relationship between snow depth and ice thickness is expected due to the thermal insulating effect of snow on ice growth [73]. Based on the variability of site-averaged FYI thicknesses, it is clear that FYI sites had formed at different times. Therefore, snow depth was rather a function of ice age: FYI that formed earlier had more time to accumulate snow, and for ice to grow, compared to newer ice. In addition, snow re-distribution and/or snow fall later in the season would have a minimal insulation effect. On MYI, snow depth and core length had a negative relationship. Apart from the thermal insulating effect, surface topography also plays an important role for snow distribution on MYI [74]. In contrast to more level FYI, MYI has an undulating surface due to accumulation of melt ponds, presence of hummocks, pressure deformation and differential melt. This subsequently promotes the accumulation of snow in thinner low spots during wind driven re-distribution, leading to the observed negative relationship between ice thickness and snow depth.

### Annual layers

The FY portions of the MYI, and all of the FYI cores were representative of ice forming locally in the Lincoln Sea during the previous winter. FY ice portions had thicknesses between 0.4 and 0.7 m, which agrees well with reported literature values for new-ice growth at the bottom of MYI between 0.45 and 0.55 m [69,73,75]. Based on general sea ice circulation patterns [59] the MYI in our region had most likely spent a substantial portion of its life within the central Arctic Ocean. Therefore the MY portions of the MYI, were likely representative of central Arctic MYI.

Annual layers in MYI cores were identified at the transition from columnar ice texture of FY portions to granular and mixed texture in the MY portion. Local chl *a* peaks were observed to coincide with all of the identified annual layers and are probably remnants from the previous year’s algal communities. A previous study [76] also observed internal chl *a* peaks that corresponded with a transition from mixed to columnar ice types, but did not conclude it was an annual layer. The correspondence of both texture and bulk salinity profiles with local chl *a* peaks and the agreement with literature values provides strong evidence that the internal chl *a* peaks corresponded to the bottom ice algae layers from previous years.

### Overview of chlorophyll *a* concentrations

A comparison of our maximum bottom-integrated chl *a* concentration (1.9 mg chl *a* m^-2^) with maximum bottom-integrated chl *a* concentrations reported for different regions during spring (see summary in [48] and references therein) shows that our chl *a* values from the Lincoln Sea were low compared to other regions of the western Arctic Ocean. In the Canadian Arctic Archipelago, values were one to two orders of magnitude higher (14 to 340 mg chl *a* m^-2^), and in the Baffin Bay and the Beaufort Sea they were over one order of magnitude higher (24 to 64 mg chl *a* m^-2^). The large majority of these and other sea ice studies, however, focus primarily on FYI [48]. Consequently a comparison with spring MYI chl *a* concentrations is limited to only a few studies. Our range of core-averaged MYI chl *a* concentrations (0.1 to 0.8 mg chl *a* m^-3^) is in agreement with other spring MYI values (~ 0.1 to 0.65 mg chl *a* m^-3^ [77]), observed during a drifting study at slightly lower latitudes (75 to 80°N) in the Beaufort and Chukchi Seas. Our FYI maximum core-averaged chl *a* concentration value (2.1 mg chl *a* m^-3^), however, was an order of magnitude higher than maximum FYI core-averaged concentrations measured during that study in April to May (~0.2 mg chl *a* m^-3^). Melnikov et al. (2002) [77] reported highest MYI chl *a* concentrations during July providing some evidence that our study was conducted during the early stages of the growth season, and maximum biomass levels had not yet been reached. Spring MYI chl *a* concentration values comparable to our study were also reported from the Bering Sea with core-integrated concentrations between 0.3 to 3.0 mg chl *a* m^-2^ [78], the Greenland Sea with core-integrated concentrations between 0.73 to 2.63 mg chl *a* m^-2^ [79], and Fram Strait with section concentrations between 0.0 to 3.4 mg chl *a* m^-3^ [49].

### Comparison of Ice Age Portions (MY, FY, FYI)

It is well documented that differences in chl *a* biomass are observed in FYI at the same location due to small scale variations in snow and ice properties (see review in [27]). We have shown that FYI and MYI have different snow and ice properties; suggesting different under-ice light regimes. Based on FYI and FY samples that have grown in the same region (i.e., similar water properties), it might be expected to observe different ranges in chl *a* concentration values between the ice ages, due to differences in the light regimes. Our observations, on the other hand, showed similar chl *a* concentrations for FYI and for FY samples, and similar bottom chl *a* concentrations for FYI and MYI. This indicates that the range of under-ice light conditions may be similar under FYI and MYI (FY), regardless of large differences in their physical properties.

It might also be expected that the upper MY portion would have had lower chl *a* concentrations than the other ice age portions, because it experienced vertical flushing of the ice column during the previous melt season(s), and had no access to nutrient replenishment from the underlying water column. However, during our study the upper MY still had chl *a* concentration values similar to FY and FYI. This observation can be attributed to the presence of remnant communities within the previous year’s bottom layers and melt ponds. Refrozen melt-ponds were identified at or near the surface of 6 out of 11 MYI cores. Each coincided with an elevated chl *a* concentration in the corresponding core section. Algal communities in surface layers of Arctic sea ice are not common. However, similar features were observed in summer sea ice by Gradinger et al., (2005), and were attributed to freshwater melt pond inhabitants [51].

Maintenance of the previous years’ algal biomass levels in the annual layers was supported by a separation of bacterial communities in one MYI core. At MYI site 1–11, distinct bacterial assemblages were observed at different depths (i.e., surface melt ponds, MY, and FY) based on analyses of the 16S rRNA gene from one coincident core [55]. This suggests that carbon sources were high enough and vertical exchange was sufficiently low to sustain different bacterial communities within the entire MYI column at site 1–11. Although the presence of sequences classified as closely related to cyanobacteria have been reported in Arctic summer pack ice [80], these sequences were not observed in the MYI core from site 1–11 [55]. This suggests that the chl *a* maxima, observed throughout the core, originate from phototrophic eukaryotes (i.e. diatoms and flagellates), which is in agreement with a previous study that reported high flagellate and diatom biomass in the upper and bottom portions, respectively, of Arctic summer ice [81].

### Chl *a-*snow/ice relationships

The relatively high bottom-integrated chl *a* concentrations at sites with the lowest snow depth and highest potential light availability indicates that the limiting factor for algal growth during this study was light availability. This is consistent with other studies that found light to be limiting algal growth during the early-spring growth season (e.g., [40,82]). The difference between landfast ice and pack ice chl *a* concentrations may also suggest a limited influence of nutrient availability on algal growth during our study.

The logistic regression analysis showed that core-normalized bottom integrated chl *a* concentrations were nearly zero at snow depths >0.17 m, or at bulk integrated extinction coefficients >5.8 (Fig 8b and 8c). Ice cores that had snow depth and bulk integrated extinction coefficients below these critical values for algal growth also had the highest bottom ice chl *a* concentrations between 6.4 and 15.4 mg m^-3^. A similar influence of snow depth on chl *a* concentration has been reported previously by using an exponential relationship, identifying a similar threshold value for snow depth on FYI [35]. The main feature of the logistic regression is the identification of a critical threshold (inflection point). The critical value divides the snow depth and bulk integrated extinction coefficient values into two conditions, either: 1) favorable for algal growth (higher chl *a*); or 2) not favorable for algal growth (lower chl *a*).

Combining the mean downwelling incoming radiation values with the critical threshold bulk integrated extinction coefficient value (5.8) as parameters in a simplified light extinction model (equation 1 in [32]), results in an estimated daily mean available irradiance for bottom ice algae of 3 μmol photons m^-2^ s^-1^. This estimate is in good agreement with reported critical minimum under-ice irradiance levels to maintain algal growth between ~2 and 9 μmol photons m^-2^ s^-1^ [83–85]. The strong relationship between snow depth and bottom-integrated chl *a* concentration emphasizes the dominating effect of snow depth on light transmission and subsequent algal growth. The combined effect of snow and ice on light transmission and subsequently on chl *a* concentration in the lowermost 0.2 m of sea ice demonstrates that both parameters should be considered when comparing ice of variable thicknesses and snow depths.

Although snow typically has a dominating effect on light transmission compared to sea ice, which has typical extinction coefficients between 0.8 and 1.55 m^-1^ (e.g., [28,32–34]), extinction coefficients of snow in the visible spectrum can vary by over an order of magnitude from 4 m^-1^ for wet snow to between 40 and 80 m^-1^ for fresh snow (e.g., [28–31]). The low extinction coefficients associated with wet snow or high coefficients associated with fresh snow are likely not representative of our study region. Air temperatures were well below freezing during the study and the fresh snow extinction coefficient, as the name implies, is an intermittent property of the snow that is not representative over longer periods. Therefore, we consider the extinction coefficient for snow of 20.0 m^-1^ used here, based on the mean snow density, a realistic estimate of snow properties for the study region. Biomass in sea ice also reduces available light for other in-ice or under-ice phototrophic organisms [35]. Using observed specific absorption coefficients for sea ice algae between 0.003 and 0.010 m^-1^[mgchl*a*m^-3^]^-1^ in the spectral range 400 to 500nm [86] would amount to absorption coefficients between 0.02 and 0.15 m^-1^, for our maximum chl *a* concentrations (6.4 to 15.4 mgm^-3^). This suggests that light limitation and self-shading by in-ice algae would have been minimal during our study.

Based on the combined effect of snow and ice on bottom integrated chl *a* concentration, it is important to address the representativeness of the ice cores in terms of the actual ice thickness and snow distribution in the Lincoln Sea. Bottom chl *a* concentrations were highest in 2 FYI cores from refrozen leads that were likely younger than the other FYI sites based on snow depth and ice thickness survey measurements. A third core (6–12) that was also from a younger refrozen lead did not have high biomass even though it was the thinnest core and had lowest snow depth. This could be explained by higher under-ice irradiance, which would have inhibited algal colonization until light levels became more favorable or until algal cells would have had sufficient time to adapt to the light conditions (e.g., [36,41,42,87]). Second, the recent or current ice growth rate may have been too rapid to establish substantial algal biomass [88].

A common feature, which influences the formation of refrozen leads and FYI, in the Lincoln Sea is an ice arch that forms at the entrance to Nares Strait [89,90]. With the presence of an ice arch and more stable ice conditions, FYI represented less than 15% of all airborne ice thickness measurements in the Lincoln Sea [53,54]. However, in the absence of an ice arch FYI represented up to 20% of all airborne ice thickness measurements due to a more mobile ice pack [53]. Under typical stable conditions in the Lincoln Sea (e.g., with the presence of an ice arch) newer refrozen leads would likely represent a smaller fraction of the overall FYI cover. This implies the 2 cores with highest bottom chl *a* concentrations were not representative of typical FYI in this region. However, in years with unstable conditions (e.g., no ice arch forming) in the Lincoln Sea and in a future Arctic system with a more mobile ice pack the relative coverage of refrozen leads will likely increase and perhaps become an increasingly more important component of overall sea ice algal biomass in the Lincoln Sea and Arctic Ocean [5].

### Underestimation of MYI Algal biomass

As discussed previously, there is a sampling bias towards thinner MYI in this and many other studies. In this study the core with the maximum MYI bottom chl *a* concentration was extracted from a hummock. The hummock core, 5–10, corresponded to the thickest core with shallowest snow depth and high chl *a* biomass (Table 3). This indicates the potential for relatively higher algal biomass in under-represented thick MYI hummocks, which typically have lower snow coverage. In a study from the Fram Strait [49], maximum spring bottom chl *a* concentrations were also observed in the thickest MYI core with lowest snow depth when compared to two other second-year ice cores.

From a total of 538 snow depth measurements conducted on MYI, 15% were below the critical snow depth value of 0.17 m (Fig 8a). This value of 15% may be a good estimate for the distribution of hummocks in the region. Due to the significantly stronger influence of snow depth than ice thickness on light attenuation, these regions of thicker ice and less snow (e.g., hummocks) could be the only regions of MYI where transmitted under-ice PAR is above a threshold value for algal growth during spring. This becomes more apparent if we consider the potential bulk integrated extinction coefficients of different ice types using a range of extinction coefficients. Based on typical snow densities in the region, we use snow extinction coefficients of 20 and 25 m^-1^ combined with the full range of reported values for ice of 0.8 and 1.55 m^-1^. These calculations result in bulk integrated extinction coefficients between 2.8 to 5.4 for a 3.5 m hummock with no snow, 4.8 to 7.9 for a 3.5 m hummock with 0.1 m of snow, and 5.6 to 8.1 for a snow covered melt pond (0.2 m of snow and 2 m of ice). Light attenuation is likely different between refrozen melt pond ice and hummocks, which is apparent when you compare the texture images for deteriorated ice (Fig 2d), typical of hummock surface ice, and melt ponds (Fig 2e). The corresponding spring time extinction coefficients are unknown for hummocks and melt ponds and therefore the full range of reported values were used to account for the potential variability. Although extinction coefficients for snow are highly variable we used an upper limit of 25 m^-1^ to account for some variability in snow properties and the influence of small amounts of fresh snow that may be intermittently present throughout the spring season.

The above mentioned ranges of extinction coefficients demonstrate that thick hummock ice, with little or no snow, has the potential for higher amounts of available under-ice PAR and more importantly bulk integrated extinction coefficients below the critical value of 5.8. Furthermore, this suggests that under similar nutrient and incoming solar radiation conditions, 15% of MYI, which has little or no snow coverage, has the potential for bottom algal layers similar to or greater than the observed maximum MYI chl *a* concentration of 6.4 mg m^-3^. This value is low compared to FYI in the Canadian Arctic Archipelago where bloom values at the ice bottom can reach concentrations greater than 100 mg m^-3^ (e.g., [35,46,91]). However, taking into account that our study was conducted during the early algal growth season, we would expect to observe more algal growth and higher chl *a* concentrations later in the season. If the observed maximum MYI value was extrapolated over 15% of the thicker MYI regions (e.g., 3+ year old MYI extent for March 2011 was >1.5 x 10^6^ km^2^ [1]), taking into account the potential for higher biomass expected during bloom, these regions could represent a substantial amount in terms of chl *a* biomass and, possibly, primary production.

### Implications for a changing Arctic (shift from MYI to FYI)

In light of the limited number of recent studies, one may argue that our current understanding of Arctic sea ice algal biomass in MYI is based on a historic Arctic that was different from today. The melt season has lengthened [6,7], and sea ice thickness, extent, and volume have undergone drastic changes [5]. MYI is disappearing from the Arctic at a rate faster than predicted by models, with a seasonally ice-free Arctic likely to occur before the end of this century, possibly as early as 2020 [10], resulting in the complete, or near complete, loss of MYI. Measurements of primary production in the central Arctic Ocean indicate that sea ice production can account for over 50% of total primary production [92], but it remains unclear how ice-associated production will change with a shift from MYI to FYI.

Arctic FYI studies in general have shown a higher range of bottom chl *a* biomass in FYI compared to MYI. FYI values include highly productive regions (e.g. Arctic shelves) where MYI is not present or has not been studied. Here we show that FYI and MYI in the Lincoln Sea can have comparable chl *a* biomass during the spring period (May). Previous studies have also demonstrated comparable or even slightly higher chl *a* biomass in MYI compared to FYI [77]. In addition, maximum chl *a* biomass values have also been observed in the thickest sea ice during spring [49] and summer [50]. Based on our results and previous studies that show generally higher, or similar, chl *a* biomass potential in MYI compared to FYI, we suggest that the general view of higher productivity in FYI than in MYI should be revisited in order to achieve a better understanding of the current and future state of the Arctic system.

If we base future estimates of ice-algal production on the fact that FYI from Arctic shelf regions is more productive than MYI in general, this would lead to the assumption that sea ice algal production would increase with a replacement of MYI by FYI during the ice-covered period. However, our results suggest only minor changes in ice algal biomass when all MYI is replaced by FYI. This considered in combination with the underestimated chl *a* biomass potential of thick MYI (hummocks) suggests the on-going loss of MYI in the Arctic Ocean may have a larger impact on ice–associated production than generally assumed.

Our results also showed that younger FYI (e.g., refrozen leads) had the highest chl *a* biomass and that the comparable values between MYI and FYI are likely driven by the higher biomass in the younger FYI. The relative proportion of younger FYI and refrozen leads in the central Arctic Ocean will likely increase with continued increases in ice drift velocities and a thinning ice pack [5,13,93]. The increase in younger FYI and refrozen leads will likely result in a general increase of ice algal biomass during the bloom period, the extent of which will depend on the spatial extent and regional variability of these features.

The expected higher bloom biomass of thinner FYI, however, may not result in a net increase in ice algal production over the entire growth season. Even with larger areas of thinner FYI, the expected increase in maximum ice-algal biomass may not compensate for the increased vulnerability of thinner ice to rapid changes in the light field and rapid snow/ice melt. These vulnerabilities could result in earlier termination of the ice-algal bloom due to photo-inhibition and/or rapid melt [36,40]. Earlier termination of the ice-algal bloom has been linked to a mismatch with the reproductive cycles of key grazers having negative consequences for the entire food web [19,47]. In addition, sea ice decline has already been linked to increased export of POC and algal aggregates to the sea floor (e.g., [20,94]), which indicates an associated removal of carbon and nutrients from surface waters. Higher carbon and nutrient export rates in the future may result in a situation where a rapid increase followed by a rapid decline in ice-associated primary production would not be sustainable for longer periods due to the removal of nutrients. This would be analogous to a boom-bust cycle. The MYI system, however, is less vulnerable to rapid environmental changes and therefore could be considered a more sustainable system where rapid sinking of ice-algae (i.e., carbon and nutrients) is less likely. Thus, the MYI system may have the potential to sustain biogeochemical cycles required to maintain moderate levels of algal biomass over longer periods (i.e., higher net primary production).

## Conclusions

Studies comparing biogeochemical properties of first-year sea ice (FYI) with multi-year sea ice (MYI) in the high Arctic are essential to understand the potential biogeochemical changes to sea ice ecosystems in a future Arctic Ocean with little or no MYI. In light of the current limited investigations of Arctic MYI algae, the present study provides a unique multi-annual dataset comparing ice-algal chl *a* and physical properties of both FYI and MYI during spring from a high-Arctic system. The low variability in chl *a* concentrations, both within and between MYI and FYI in the coastal Arctic Ocean, suggests little or no change in algal biomass with a shift from MYI to FYI. The apparent relationship between chl *a* biomass in the bottom layer of ice and bulk integrated extinction coefficients of the snow-ice matrix, implies that an appropriate representation of areas with low snow depths, such as MYI hummocks, is critical for a realistic estimation of the MYI contribution to overall ice algal biomass estimates in the Arctic Ocean. The potential for higher ice algal biomass in thick MYI with less snow, in conjunction with a lack of significant difference between FYI and MYI chl *a* biomass during our study suggests that the on-going loss of MYI in the Arctic Ocean may have a more negative impact on ice–associated production than generally assumed.
